# Electrogenic Binding of Intracellular Cations Defines a Kinetic Decision Point in the Transport Cycle of the Human Serotonin Transporter*

**DOI:** 10.1074/jbc.M116.753319

**Published:** 2016-10-18

**Authors:** Peter S. Hasenhuetl, Michael Freissmuth, Walter Sandtner

**Affiliations:** From the Institute of Pharmacology, Center of Physiology and Pharmacology, Medical University Vienna, Vienna, Waehringerstrasse 13a, A-1090 Vienna, Austria

**Keywords:** electrophysiology, kinetics, monoamine transporter, neurotransmitter transport, serotonin

## Abstract

The plasmalemmal monoamine transporters clear the extracellular space from their cognate substrates and sustain cellular monoamine stores even during neuronal activity. In some instances, however, the transporters enter a substrate-exchange mode, which results in release of intracellular substrate. Understanding what determines the switch between these two transport modes demands time-resolved measurements of intracellular (co-)substrate binding and release. Here, we report an electrophysiological investigation of intracellular solute-binding to the human serotonin transporter (SERT) expressed in HEK-293 cells. We measured currents induced by rapid application of serotonin employing varying intracellular (co-)substrate concentrations and interpreted the data using kinetic modeling. Our measurements revealed that the induction of the substrate-exchange mode depends on both voltage and intracellular Na^+^ concentrations because intracellular Na^+^ release occurs before serotonin release and is highly electrogenic. This voltage dependence was blunted by electrogenic binding of intracellular K^+^ and, notably, also H^+^. In addition, our data suggest that Cl^−^ is bound to SERT during the entire catalytic cycle. Our experiments, therefore, document an essential role of electrogenic binding of K^+^ or of H^+^ to the inward-facing conformation of SERT in (*i*) cancelling out the electrogenic nature of intracellular Na^+^ release and (*ii*) in selecting the forward-transport over the substrate-exchange mode. Finally, the kinetics of intracellular Na^+^ release and K^+^ (or H^+^) binding result in a voltage-independent rate-limiting step where SERT may return to the outward-facing state in a KCl- or HCl-bound form.

## Introduction

Transport of solutes across biological membranes is essential to cell survival, and the underlying principles have been highly conserved during phylogeny (1); all transporters operate via alternating access of the substrate and co-substrate binding sites to the extracellular and the intracellular milieu. The structural rearrangements, which support this alternating access and sequentially seal off the intracellular and the extracellular translocation pathway, differ among various transporter families (2). Likewise, transporters harness distinct energy sources to complete their transport cycle. Secondary-active solute carriers (SLCs)^2^ of the LeuT superfamily, named after the eponymous leucine transporter of the thermophilic bacterium *Aquifex aeolicus*, share a common fold of two inverted repeats consisting of five transmembrane segments each. The majority of these transporters utilize the electrochemical gradient of Na^+^ to drive intracellular accumulation of the cognate substrate (2). The principal mechanisms underlying substrate translocation are thought to be conserved in this family. However, differences and uncertainties exist with respect to the nature and the number of co-transported and counter-transported ions (1). It has also remained enigmatic how transporters of the LeuT superfamily afford the energetically unfavorable translocation of the charged solutes through the electric field of the hydrophobic cell membrane. Several mechanisms may exist; for example, Cl^−^ (or a glutamate residue in the bacterial transporters) may provide the negative charge necessary for balancing the charge of a cation (*e.g.* Na^+^; Refs. 3 and 4). In addition, the overall translocated charge of a co-substrate may be distributed over several partial reactions (5). This reduces the apparent valence and thus the voltage dependence of the individual partial reactions and allows for the translocation of the charged (co-)substrates across the membrane electric field. These mechanisms are of particular relevance for those transporters, which are expressed in excitable cells, where transient voltage changes may affect substrate uptake. The transporters for the monoamines serotonin/5-HT (SERT, SLC6A4), dopamine (DAT, SLC6A3), and norepinephrine (SLC6A2), for instance, are mainly expressed in neurons, where they maintain cellular monoamine stores during neuronal firing. The monoamine transporters display two modes of action as follows.

In the forward-transport mode, the transporter releases its substrate and co-substrates into the cytosol and completes the catalytic cycle by returning from the inward-facing conformation in an empty state or may countertransport other co-substrates (*e.g.* K^+^). This results in vectorial substrate uptake.

In addition, the transporter can enter a substrate-exchange mode; in this mode it switches between the substrate-bound outward-facing and the substrate-bound inward-facing conformations without completing its catalytic cycle. The transporter may translocate the extracellular substrate into the cell and return with an alternative substrate originating from the cytosol. This results in release of the intracellular substrate. This mode is the basis for the action of amphetamines (6). Alternatively, the transporter may subsequently return to the outward-facing conformation loaded with the same substrate molecule because substrate dissociation from the inward-facing conformation is blocked. This precludes uptake and is physiologically undesirable.

The switch between these transport modes and the key variables, which define this decision point, can only be understood, if intracellular (co-)substrate binding and release is addressed by time-resolved measurements. Here, we exploited the fact that translocation of substrate and co-substrates and the forward-transport mode give rise to characteristic currents through SERT. We used the whole-cell patch clamp technique to study the binding of intracellular Na^+^ and Cl^−^ and of the counter-transported cations K^+^ and H^+^ to the inward-facing conformation of SERT. The experiments identify a crucial role of electrogenic cation binding in controlling the transport modes of SERT.

## Results

### 

#### 

##### Substrate-induced Currents as Probes for Individual Partial Reactions of SERT

**FIGURE 1.**
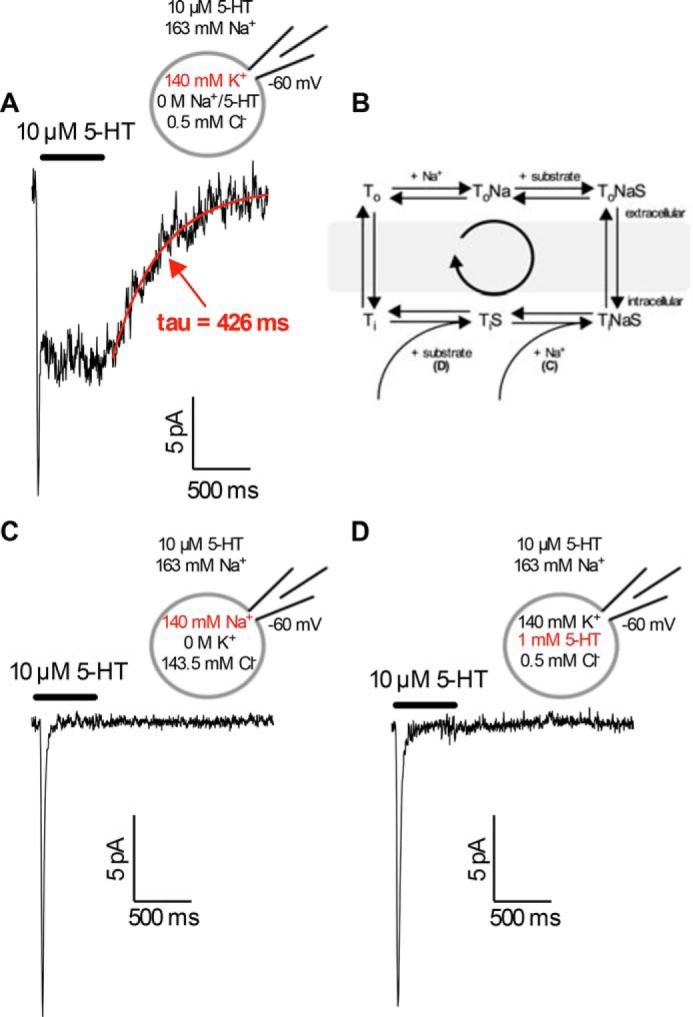
**Peak and steady-state current observed with different internal solutions.** *A*, representative 5-HT-induced current recorded from a HEK-293 cell expressing SERT in the presence of physiological ion gradients. Note that the current comprises two components: a peak current and a steady-state current. The steady-state current relaxes upon washout of 5-HT. The time-course of relaxation was fitted with a monoexponential function (also see Fig. 7*B*). *B*, kinetic scheme of a Na^+^-coupled transporter. *C* and *D*, the use of 140 mm Na_*i*_^+^ (*panel C*) or 1 mm 5-HT*_i_* (K^+^ 140 mm; *panel D*) isolates the peak current from the steady-state current by eliminating the latter. Cells were clamped to −60 mV, and external 5-HT (10 μm) was applied for 500 ms. The traces shown are representative of at least 10 independent experiments.

The whole-cell patch clamp technique is useful to probe intracellular binding reactions because it allows for control of intracellular reactants and voltage and provides high temporal resolution (7). Substrate translocation by transporters of the SLC6 family has long been known to be associated with currents (8–10). SERT is thought to display an electroneutral stoichiometry (11). Nevertheless, substrate application elicits an uncoupled Na^+^ conductance (10, 12). Recently, the currents through SERT have been reconciled with a kinetic model of its catalytic cycle (13). Based on this model, the two current components arising upon rapid 5-HT application to SERT-expressing HEK-293 cells can serve as signals to probe individual partial reactions during 5-HT transport (Fig. 1, *A* and *B*) (14, 15) as follows.

The peak current reflects the initial movement of substrate and co-substrates through the electric field of the membrane. Substrate-induced peak currents are not unique to SERT; they have also been recorded for DAT (16). It has, however, remained unclear which partial reaction carries the charge that gives rise to this current.

The steady-state current corresponds to the aforementioned uncoupled Na^+^ conductance (10, 12). This conducting state is in equilibrium with a K^+^-bound inward-facing conformation. SERT visits the conducting state during the transition from the inward-facing to the outward-facing conformation (13).

**FIGURE 2.**
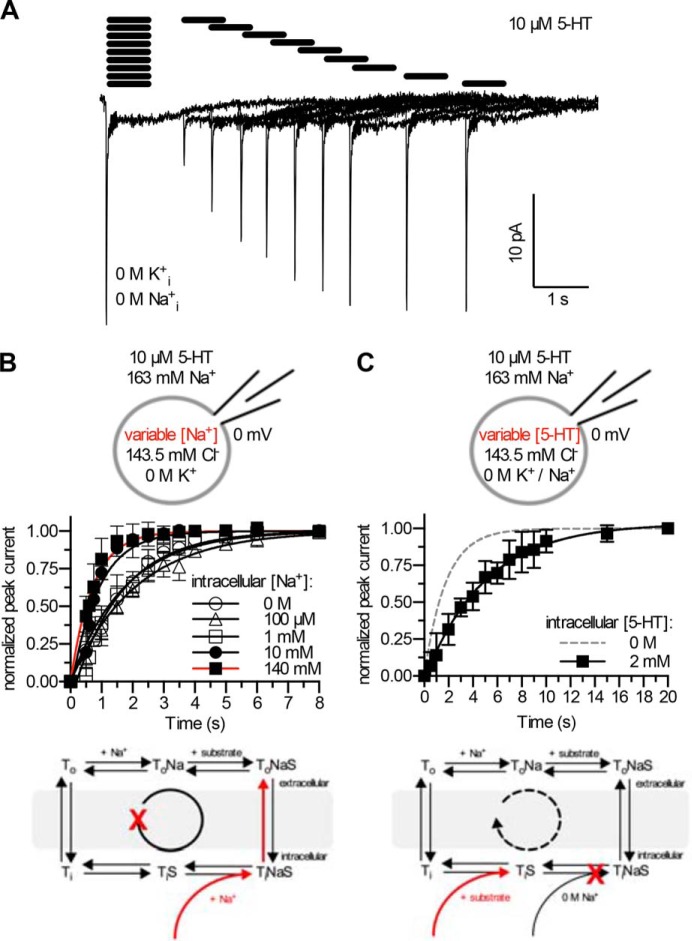
**Actions of intracellular Na^+^ and 5-HT.** *A*, peak current recovery protocol and representative traces. 5-HT (10 μm) was applied for 500 ms followed by variable (increasing) washout intervals and subsequent 5-HT test pulses. The current amplitude elicited by 5-HT reads out the fraction of transporters available for 5-HT binding and, thus, recovery to the outward-facing conformation. The time course of peak current recovery was fitted to a monoexponential function, yielding the recovery rates (*k_r_*) shown in *panels B* and *C. B*, the rate of peak current recovery *k_r_* as a function of [Na^+^]*_i_* in a K^+^-free solution at 0 mV. 0 m Na_*i*_^+^ (*n* = 7): 0.568 s^−1^ [0.532 − 0.604]; 100 μm Na_*i*_^+^ (*n* = 5): 0.528 s^−1^ [0.463 − 0.593] 1 mm Na_*i*_^+^ (*n* = 7): 0.628 s^−1^ [0.567 − 0.689]; 10 mm Na_*i*_^+^ (*n* = 10): 1.138 s^−1^ [1.039 − 1.238]; 140 mm Na_*i*_^+^ (*n* = 11): 1.408 s^−1^ [1.260 − 1.557]; numbers in *brackets* denote the 95% confidence interval. Data are the means ± S.D. *Scheme*: the dependence of *k_r_* on [(co-)substrates]*_i_* allows for probing of intracellular binding reactions because the recovery from the inward- to the outward-facing conformation is a function of intracellular binding reactions, *e.g.* binding of Na_*i*_^+^. Assuming a sequential order where Na^+^ dissociates before the substrate, Na^+^ rebinding induces the substrate-exchange mode (*red arrows*). *C*, internal solution: pH 7.2, 0 m Na^+^, 0 m K^+^, 143.5 mm Cl^−^, 2 mm 5-HT. Data of peak current recovery were fitted to a monoexponential function to obtain the peak current recovery rate *k_r_* and compared with the data shown in *panel B* (the *dashed line* represents the fit of the 0 m Na_*i*_^+^ condition). A 5-HT*_i_* concentration of 2 mm reduces *k_r_*: 0.202 s^−1^ [0.160 − 0.243]. Data are the means ± S.D. (*n* = 7); *numbers in brackets* denote the 95% confidence interval. Note the different scaling of the *x* axis in *panels B* and *C. Scheme*: binding of 5-HT*_i_*. Assuming a sequential order where Na^+^ dissociates before the substrate, rebinding of 5-HT*_i_* fails to induce the substrate-exchange mode but delays the forward-transport rate (*red* and *dashed arrows*).

We used these two current components to study intracellular (co-)substrate binding. Dissociation of Na^+^ (from the conserved Na_2_-site) is thought to trigger conformational changes essential for substrate dissociation from transporters that share the LeuT fold (17–24). This supports progression through the transport cycle. Increasing intracellular Na^+^ or substrate concentrations ([Na_*i*_^+^/5-HT*_i_*]) precludes this progression. The steady-state current requires completion of the catalytic cycle. Accordingly, rebinding of intracellular (co-)substrates is predicted to eliminate the steady-state current (16, 25, 26). The suppression of the steady-state current was readily detectable in the presence of high intracellular levels of Na^+^ (Fig. 1*C*) and 5-HT (Fig. 1*D*). However, elimination of the steady-state current does not provide any information on the kinetics, voltage dependence, or the order of intracellular solute binding. We, therefore, studied intracellular co-substrate binding in more detail using the peak current as signal (Fig. 2*A*).

##### Binding of Intracellular Na^+^ and 5-HT to the Inward-facing Conformation of SERT

We first focused on intracellular binding of Na^+^ by relying on a protocol, where the brief application of 5-HT was followed by a second pulse of substrate after a defined interval. This allowed for recording the time course of peak current recovery (*cf*. original traces in Fig. 2*A*).

The amplitude of this peak current is a read-out of binding sites that are available for binding of extracellular 5-HT. Recovery of the peak current amplitude is contingent on (renewed) binding of 5-HT to the outward-facing conformation and hence requires a return from the inward-facing to the outward-facing conformation of the transporter (*cf. reaction schemes* in Fig. 2). Hence, the rate of peak current recovery, *k_r_*, is a function of several partial reactions. These depend on the intracellular concentrations of substrate and co-substrates. Accordingly, measuring *k_r_* as a function of intracellular (co-)substrate concentrations allows for inferring the reaction order, the rate constants, and the voltage dependence of intracellular solute binding to SERT.

Na^+^ binding to transporters, which share the LeuT fold, is assumed to follow a “first-on/first-off” mechanism (2). This entails that binding of extracellular Na^+^ stabilizes the transporter in an outward-facing conformation, which is conducive to subsequent substrate binding. After substrate binding and translocation, the transporter releases Na^+^ from the inward-facing conformation, which allows for substrate dissociation. If this is the case in SERT, Na_*i*_^+^ re-association to the inward-facing conformation is predicted to induce the substrate-exchange mode because it precludes dissociation of 5-HT*_i_*. In contrast, increasing [5-HT]*_i_* should fail to do so (compare the *red arrows* in the *reaction schemes* in Fig. 2, *B* and *C*). In the absence of Na_*i*_^+^ (0 m, *empty circles* in Fig. 2*B*) and in the presence of 0.1 mm and 1 mm Na_*i*_^+^ (*empty triangles* and *squares*, respectively, in Fig. 2*B*), the rate of peak current recovery *k_r_* was virtually identical, suggesting that these concentrations allowed for completion of the transport cycle in the forward-transport mode. Conversely, peak current recovery was accelerated in the presence of 140 mm Na_*i*_^+^ (*closed squares* in Fig. 2*B*). This observation was consistent with the interpretation that rebinding of Na_*i*_^+^ to the inward-facing conformation had induced the substrate-exchange mode (*cf. reaction scheme* in Fig. 2*B*). The time course of recovery was already close to maximum at 10 mm Na_*i*_^+^ (*closed circles* in Fig. 2*B*). In contrast, 2 mm 5-HT*_i_* reduced the rate of peak current recovery (Fig. 2*C*; note the different scaling of the *x* axis). This can be rationalized by taking into account that intracellular re-binding of 5-HT slows down progression through the transport cycle (*cf. reaction scheme* in Fig. 2*C*). More importantly, it can also be inferred that, contrary to Na_*i*_^+^, high intracellular 5-HT failed to induce the substrate-exchange mode (*cf. reaction schemes* in Fig. 2, *B* and *C*). These observations suggest a sequential binding order where dissociation of Na_*i*_^+^ from the inward-facing conformation of SERT occurs before substrate release.

##### Voltage-dependent Binding of Intracellular Na^+^

**FIGURE 3.**
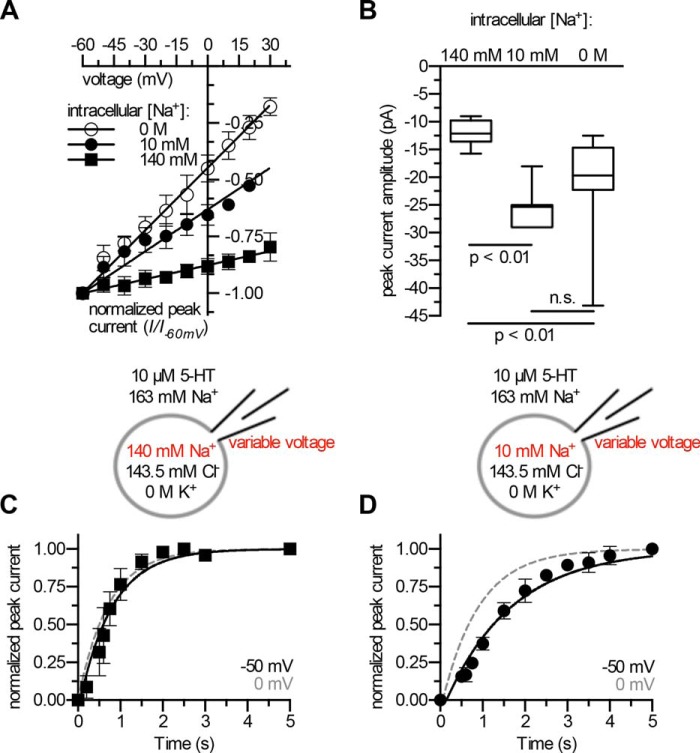
**Intracellular Na^+^ unbinding is electrogenic.** *A*, the voltage dependence of the transient peak current is contingent on [Na^+^]*_i_* (*n* = 6–7): increasing [Na^+^]*_i_* reduces the voltage dependence of the peak current. All solutions were K^+^-free. Data were fitted by linear regression. *B*, amplitude of substrate-induced peak current at intracellular Na^+^ concentrations of 0 m (*n* = 16; −mean: 20.64 pA; median: −19.70 pA; 95% confidence interval (CI): −24.93 − −16.34 pA), 10 mm (*n* = 7; mean: −25.33 pA, median: −25.30 pA; 95% CI: −28.73 − −21.92 pA) and 140 mm (*n* = 9; mean: −12.00 pA, median: −12.10 pA; 95% CI: −13.73 − −10.27 pA) at 0 mV. Statistically significant differences were determined by a Kruskal-Wallis test followed by Dunn's multiple comparisons test. Data are presented as *box plots* showing the median and the interquartile range; *whiskers* indicate minimum and maximum values. *n.s.*, not significant. *C* and *D*, the rate of peak current recovery *k_r_* is dependent on voltage, and [Na^+^]*_i_*: *k_r_* was determined at −50 mV and compared with *k_r_* at 0 mV (*dashed lines* represent the fitted curves recorded at 0 mV in the presence of the corresponding [Na_*i*_^+^] and taken from Fig. 2*B*); *k_r_* at −50 mV was not affected in the presence 140 mm (*n* = 7; *panel B*) internal Na^+^: *k_r_* 1.266 s^−1^ [1.096 − 1.436]. However, at a [Na^+^]*_i_* of 10 mm (*n* = 7; *panel C*), *k_r_* was reduced: 0.6268 s^−1^ [0.5774 − 0.6762]; numbers in *brackets* denote the 95% confidence interval. Data are the means ± S.D.

We further examined the binding reaction of Na_*i*_^+^ by recording the voltage dependence of the peak current as a function of [Na^+^]*_i_*. As outlined above, this current is associated with the translocation of substrate and co-substrates across the membrane. This can be appreciated from the finding that the steady-state current was eliminated, but the peak current persisted, if progression through the transport cycle was precluded (*cf*. Fig. 1, *C* and *D*). However, it is not clear which partial reaction(s) generated this peak current. If the event of Na_*i*_^+^-dissociation from the inward-facing conformation carried charge, desaturation of the Na^+^ binding site by reducing [Na^+^]*_i_* ought to increase the movability of Na^+^ through the electric field. Accordingly, the voltage dependence is predicted to be contingent on [Na^+^]*_i_*. This was the case: the slope of the current-voltage relationship was inversely related to the intracellular concentration of Na^+^; it was steep in the nominal absence of Na_*i*_^+^ and progressively decreased in the presence of 10 and 140 mm Na_*i*_^+^ (Fig. 3*A*). Moreover, at 140 mm Na_*i*_^+^ the amplitude of the peak current was significantly smaller than at 10 and 0 mm (Fig. 3*B*). These data indicate that dissociating Na_*i*_^+^ ions carry the majority of the peak current and that Na_*i*_^+^ release from the inward-facing conformation is electrogenic.

If dissociation of Na_*i*_^+^ from the inward-facing conformation was indeed electrogenic (*i.e.* voltage-dependent), re-association of Na_*i*_^+^ and, thus, induction of the substrate-exchange mode must also be voltage-dependent. Moreover, this voltage dependence must also decrease with increasing [Na^+^]*_i_*. We tested this prediction by measuring the rate of peak current recovery *k_r_* using 140 and 10 mm Na_*i*_^+^ at −50 mV and comparing the rates with the corresponding *k_r_*-values observed at 0 mV. As shown in Fig. 3, *C* and *D*, negative voltage reduced *k_r_* in the presence of 10 mm Na_*i*_^+^ but not of 140 mm Na_*i*_^+^. Hence, voltage-dependent binding of intracellular Na^+^ specifies the transport mode: in its absence, SERT is poised to complete the transport cycle, but binding of Na^+^ at depolarized membrane potential drives SERT into the substrate-exchange mode.

##### Voltage-independent Binding of Substrate and Co-substrates to the Outward-facing Conformation of SERT

**FIGURE 4.**
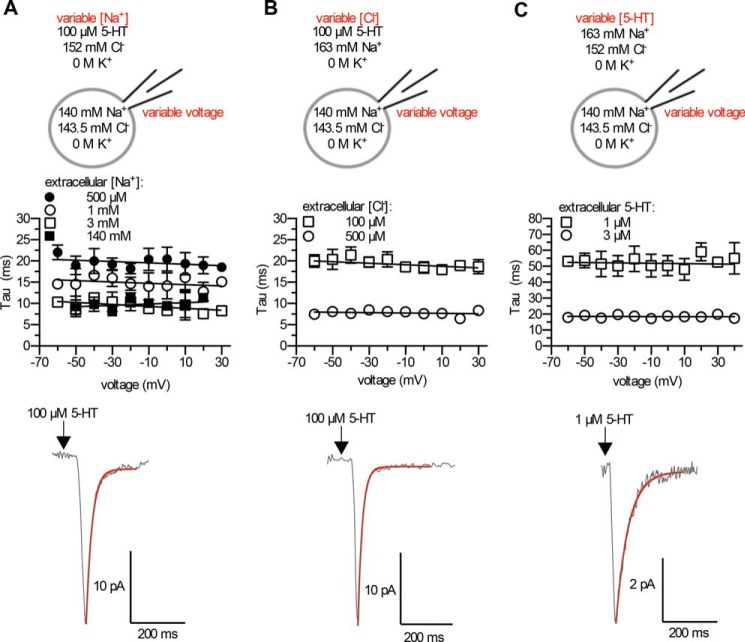
**Voltage-independent binding of extracellular Na^+^, Cl^−^, and 5-HT to the outward-facing conformation of SERT.** *A*, *top*, schematic rendering of the experimental conditions; saturating concentrations of 5-HT (100 μm) and Cl^−^ (152 mm) were used, and Na^+^ concentrations were titrated until Na^+^ binding became rate-limiting for peak current relaxation (shown in the *middle f*or 0.5, 1, 3, and 140 mm). *Middle*, the rate of peak current relaxation plotted against voltage; when extracellular Na^+^ concentrations of 0.5, 1, 3, and 140 mm were used, peak current relaxation was independent of voltage. Data are the means ± S.D. (*n* = 4–6) and were fitted by a linear regression. *Lower*, representative trace at 0.5 mm Na^+^, a concentration where Na^+^ binding was rate-limiting for peak-current relaxation. The trace is representative of six independent experiments. *B*, *panel*, schematic rendering of the experimental conditions; saturating [5-HT]*_e_* (100 μm) and [Na^+^]*_e_* (163 mm) were used, and [Cl^−^]*_e_* concentrations were titrated until Cl^−^ binding became rate-limiting for peak-current relaxation (shown in the *middle* for 0.1 and 0.5 mm). *Middle*, the rate of peak current relaxation plotted against voltage; when extracellular Cl^−^ concentrations of 0.5 and 0.1 mm were used, peak current relaxation was independent of voltage. Data are the means ± S.D. (*n* = 6) and were fitted by a linear regression. *Lower*, representative trace at 100 μm Cl^−^, a concentration where Cl^−^ binding was rate-limiting for peak current relaxation. The trace is representative of six independent experiments. *C*, schematic of experimental conditions; saturating concentrations of Na^+^ (163 mm) and Cl^−^ (152 mm) were used, and 5-HT concentrations were titrated until binding of 5-HT became rate-limiting for peak current relaxation (shown in the *middle* for 1 and 3 μm). *Middle*: the rate of peak current relaxation plotted against voltage; when extracellular 5-HT concentrations of 1 and 3 μm were used, peak current relaxation was independent of voltage. Data are the means ± S.D. (*n* = 4–7) and were fitted by linear regression. *Lower*: representative trace at 1 μm 5-HT, a concentration where 5-HT binding was rate-limiting for peak current relaxation. The trace is representative of seven independent experiments.

The inverted repeats (TM1 to TM5 and TM6 to TM10) give rise to an internal pseudosymmetry within SERT. It was, therefore, conceivable that binding of extracellular Na^+^ to the outward-facing conformation of SERT was also voltage-dependent. In fact, this was observed for the structurally related GABA transporter 1 (GAT1; Ref. 25). At saturating (co-)substrate concentrations, the relaxation rate of the peak current is limited by a conformational change. If binding of either of the (co-)substrates was voltage-dependent, the rate of peak current relaxation should change with voltage at concentrations where binding of the pertinent (co-)substrate becomes rate-limiting. We, therefore, measured the rate of peak current relaxation at concentrations where binding of either Na_*e*_^+^, 5-HT*_e_* or Cl_*e*_^−^ was rate-limiting for relaxation at voltages ranging from −60 to +30 mV. We failed to detect any voltage-dependent change in the binding of Na^+^, Cl^−^, or of 5-HT to the outward-facing conformation (Fig. 4, *A–C*).

##### H^+^ Can Functionally Replace K^+^ to Support the Steady-state Current

**FIGURE 5.**
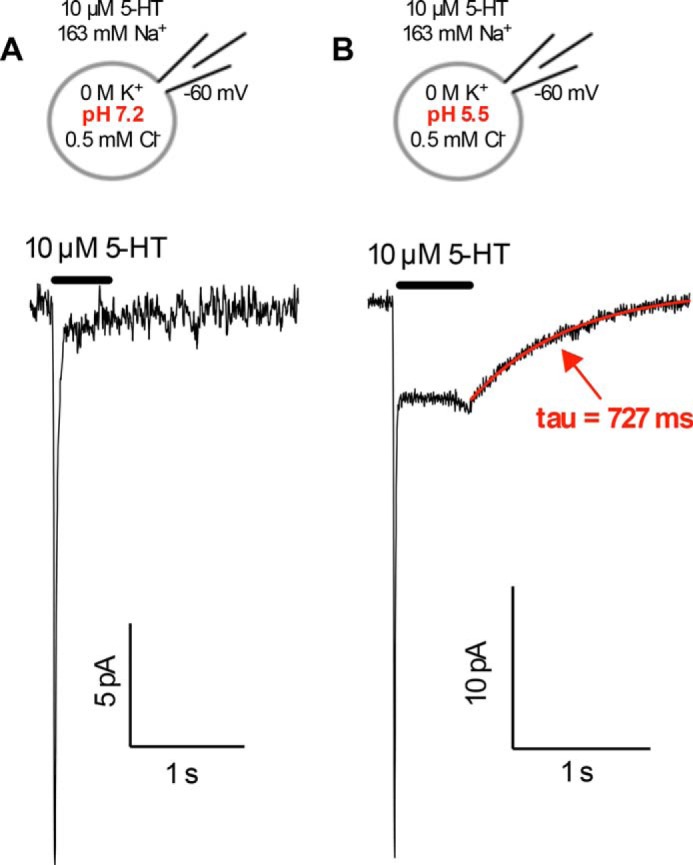
**Induction of the conducting state by intracellular K^+^ (*A*) and H^+^ (*B*).** *A*, representative current trace at pH 7.2 in the absence of internal K^+^ showing that application of 10 μm external 5-HT elicited only a peak current. Internal solution: pH 7.2, 0 m Na^+^, 0 m K^+^, 500 μm Cl^−^; no steady-state current was observed upon application of 10 μm 5-HT. *B*, representative current trace at pH 5.5 in the absence of internal K^+^ showing that application of 10 μm external 5-HT elicited both the peak and the steady-state current, which relaxed after washout of 5-HT (see Fig. 7*B* for quantification). Internal solution: pH 5.5, 0 m Na^+^, 0 m K^+^, 500 μm Cl^−^; 10 μm 5-HT elicited a steady-state current. Cells were clamped to −60 mV. The traces are representative of 10 independent experiments.

Taken together, the data summarized in Figs. 2–4 imply that the full charge of Na^+^ is moved through the membrane electric field during the intracellular unbinding reaction. This feature is unexpected because it renders 5-HT uptake vulnerable to transient changes in membrane potential and in local [Na^+^]*_i_* unless a mechanism exists that precludes re-association of Na_*i*_^+^ to the inward-facing conformation. This candidate mechanism may rely on other ions, which also bind to the inward-facing conformation but allow the catalytic cycle to proceed in the forward mode. SERT can antiport K^+^ (27). In addition, it has been suggested that H^+^ can functionally replace K^+^ (28). However, it is not clear whether these cations also fulfill a pre-steady-state (*i.e.* kinetic) function in addition to their thermodynamic role. We exploited the fact that SERT required K_*i*_^+^ to reach the conducting state (13). We first determined whether H_*i*_^+^ could functionally replace K_*i*_^+^ and recorded substrate-induced currents using K^+^-free internal solutions containing either a pH of 7.2 or 5.5. Raising [H^+^]*_i_* resulted in the appearance of 5-HT-induced steady-state currents (Fig. 5, *A* and *B*). These data imply that H^+^ ions can functionally replace K^+^ to support induction of the steady-state current.

##### Voltage-dependent Binding of K^+^ or H^+^ Supports Voltage-independent Turnover

**FIGURE 6.**
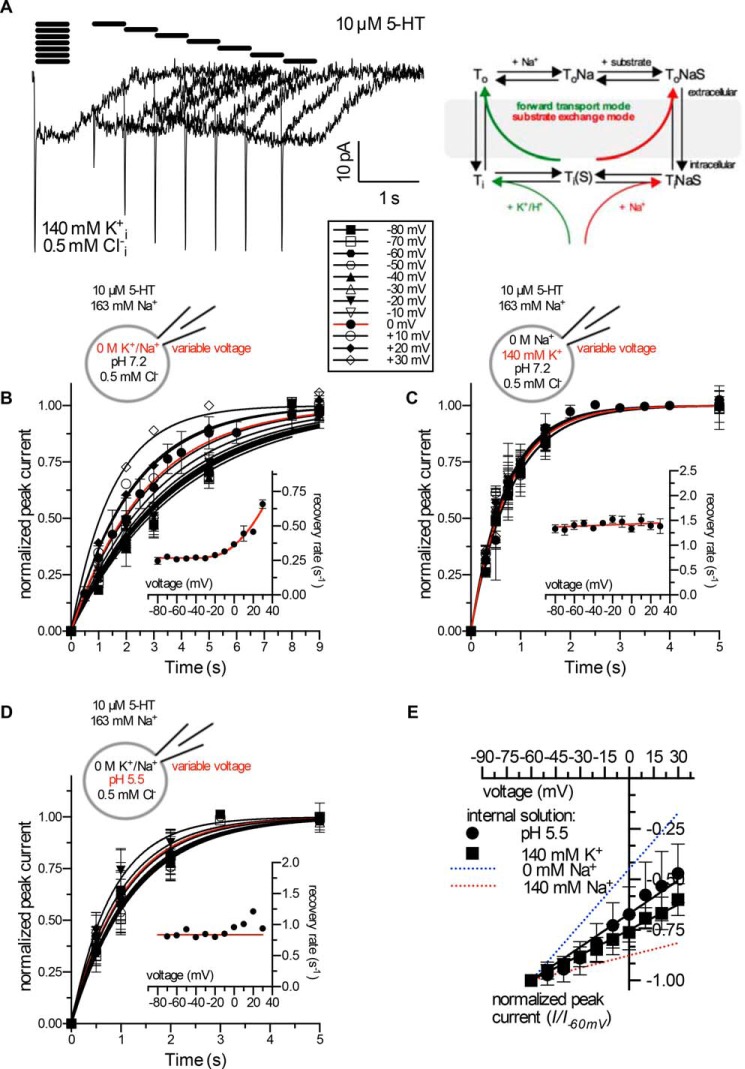
**Voltage-dependent and -independent peak current recovery in the absence and presence of internal K^+^ and H^+^, respectively.** *A*, peak current recovery protocol and representative traces using a [K^+^]*_i_* of 140 mm. *Scheme*: kinetic scheme illustrating that binding of K_*i*_^+^ or of H_*i*_^+^ may support progression through the transport cycle. *B–D*, the rate of peak current recovery *k_r_* was determined at voltages ranging from −80 mV to +30 mV; *B*, internal solution: pH 7.2, 0 m Na^+^, 0 m K^+^, 500 μm Cl^−^; data of peak current recovery were fitted to a monoexponential function. The *k_r_* values obtained from the fits were plotted as function of voltage. Data are the means ± S.D. (*n* = 5–8). *C*, internal solution: pH 7.2, 0 m Na^+^, 140 mm K^+^, 500 μm Cl^−^; data of peak current recovery were fitted to a monoexponential function to obtain estimates of the recovery rate *k_r_*. The *k_r_*-values obtained from the fits were plotted as function of voltage. Data are the means ± S.D. (*n* = 5–12). *D*, internal solution: pH 5.5, 0 m Na^+^, 0 m K^+^, 500 μm Cl^−^; data of peak current recovery were fitted to a monoexponential function to obtain estimates of the recovery rate *k_r_*. The *k_r_* values obtained from the fits were plotted as function of voltage. Data are the means ± S.D. (*n* = 5–7). *E*, voltage dependence of the peak current reduces with increasing [K^+^]*_i_* or [H^+^]*_i_*. The *blue* and *red lines* are the fits of data shown in Fig. 3*A*. Data were fitted by linear regressions. Data are the means ± S.D. (*n* = 14).

We investigated the action of K^+^ and H^+^ on the inward-facing conformation by determining the recovery rate *k_r_* in cells clamped to voltages ranging from −80 to +30 mV, thus covering the entire physiologically relevant voltage-range (*cf*. representative current traces in Fig. 6*A* and the *reaction scheme*). When a K^+^-free internal solution at pH 7.2 was used, *k_r_* was voltage-independent at negative potentials but increased at positive potentials (Fig. 6*B*). The increase in *k_r_* at positive potentials is consistent with voltage-dependent Na_*i*_^+^ re-association and the concomitant induction of the substrate-exchange mode (*cf*. the *reaction scheme* in Fig. 6). Most notably, when [K^+^]*_i_* (Fig. 6*C*) or [H^+^]*_i_* (Fig. 6*D*) was raised, the recovery rate *k_r_* was enhanced, and transporter turnover became voltage-independent. These data indicate that K_*i*_^+^ or H_*i*_^+^ and Na_*i*_^+^ bind to the inward-facing conformation in a mutually exclusive fashion. If this results from an interaction with the same (or highly overlapping) binding site(s), the association of K_*i*_^+^ or of H_*i*_^+^ requires them to pass the electric field. Hence, this reaction must also be voltage-dependent. Accordingly, binding of K_*i*_^+^ or of H_*i*_^+^ to the inward-facing conformation is predicted to reduce the electrogenicity of Na_*i*_^+^ dissociation and thus reduce the voltage dependence of the peak current. Indeed, adding 140 mm K^+^ to the pipette solution or increasing [H^+^]*_i_* to a pH of 5.5 reduced the voltage dependence of the peak current (Fig. 6*E*). These data suggest that K^+^ or H^+^ bind the inward-facing conformation of SERT in a voltage-dependent fashion and thereby blunt the voltage dependence of Na^+^ dissociation.

##### Intracellular Cl^−^ Does Not Reduce the Turnover-rate of SERT

**FIGURE 7.**
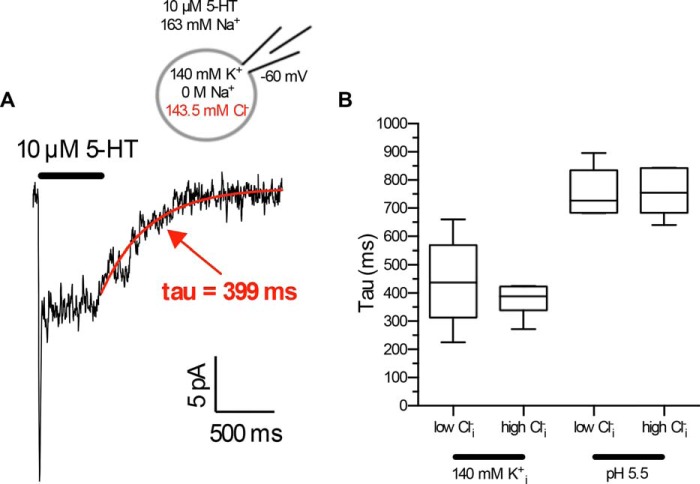
**Increasing the intracellular Cl^−^ concentration does not affect the steady-state current.** *A*, representative trace obtained at [Cl^−^]*_i_* of 143.5 mm showing that [Cl^−^]*_i_* does not eliminate the steady-state current (compare with Fig. 1*A*). The steady-state current relaxes upon washout of 5-HT. The time-course of relaxation was fitted with a monoexponential function. *B*, increasing [Cl^−^]*_i_* does not affect the relaxation kinetics of the steady-state current. 140 mm K^+^/500 μm Cl^−^ (*n* = 9), tau: mean 441.2 ms; median 437.8 ms, 95% confidence interval (CI) 325.8 − 556.6 ms; 140 mm K^+^/143.5 mm Cl^−^ (*n* = 7), tau: mean 374.1 ms, median 389.1 ms, 95% CI: 323.9 − 424.3 ms. Relaxation of the steady-state current in the presence of high [H^+^]*_i_* (*cf*. Figs. 1*A* and 5*B*): pH 5.5/500 μm Cl^−^ (*n* = 7), tau: mean 761.2 ms median: 727.0 ms, 95% CI 684.8 − 837.6 ms; pH 5.5/143.5 mm Cl^−^ (*n* = 6), tau: mean 755.5 ms, median 755.1 ms, 95% CI 670.4 − 840.7. Data are presented as *box plots* showing the median and the interquartile range; *whiskers* indicate minimum and maximum values.

As mentioned above, an additional mechanism by which the LeuT superfamily may balance the positive charge of a Na^+^ ion is the negative charge of Cl^−^ or, in the bacterial homologs, that of a glutamate residue (3, 29). The negative charge provided by the glutamate residue has been shown to undergo a transport-associated cycle of protonation and deprotonation (4). In addition, DAT, GAT1, and the intestinal glucose transporter (SGLT1 (sodium-dependent glucose transporter 1)) bind Cl^−^ but do not rely on the chloride gradient as the energy source for uptake (16, 30). SERT requires extracellular Cl^−^ (31, 32), but it has remained a matter of debate whether substrate transport is coupled to its electrochemical gradient. In a seminal study using platelet preparations, Nelson and Rudnick (31) showed that raising [Cl^−^]*_i_* to 200 mm reduced steady-state 5-HT uptake (10 min) by ∼50% compared with Cl_*i*_^−^-free conditions, but the initial transport-rate (10 s) was not affected. In contrast, in platelets preloaded with saturating concentrations of Na_*i*_^+^, both initial and steady-state uptake was completely suppressed (31). Consistent with these early findings, we observed that, in contrast to high [Na^+^]*_i_* and [5-HT]*_i_*, 143.5 mm Cl_*i*_^−^ neither eliminated the steady-state current nor affected its time-course of relaxation upon washout of serotonin (Fig. 7, *A* and *B*, also compare with Fig. 1, *A*, *C*, and *D*). These findings document that high concentrations of Cl_*i*_^−^ do not reduce the turnover rate of SERT. Two explanations can account for this, (*i*) the affinity of Cl^−^ for the inward-facing conformation is very low (hundreds of mm), and (*ii*) Cl^−^ remains bound to SERT during the entire transport cycle.

**FIGURE 8.**
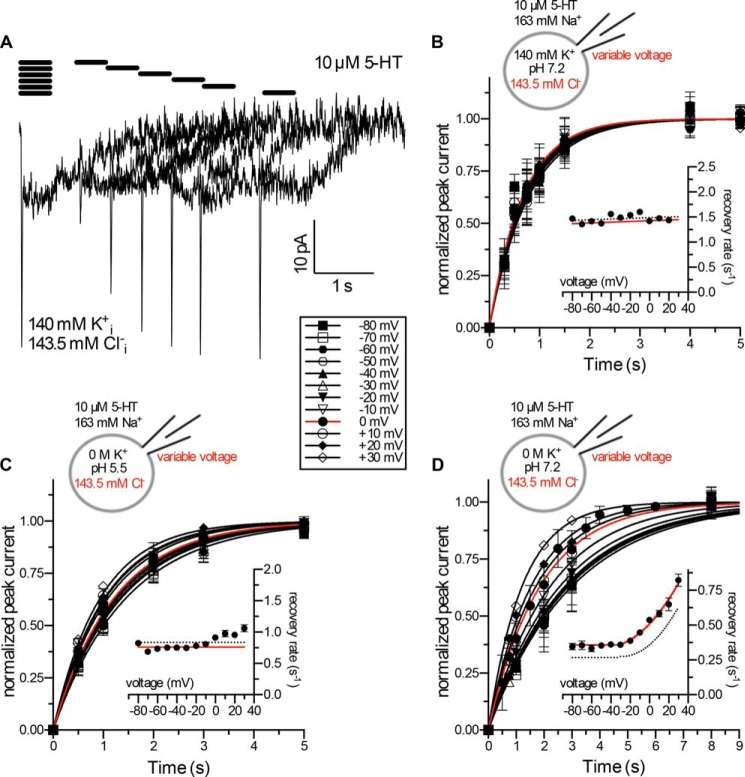
**Effect of intracellular Cl^−^ on the turnover rate of SERT.** *A*, representative current traces of the peak current recovery protocol using a [K^+^]*_i_* of 140 mm and a [Cl^−^]*_i_* of 143.5 mm. *B–D*, the rate of peak current recovery *k_r_* was determined at voltages from −80 mV to +30 mV. Data are the means ± S.D. *B*, internal solution: pH 7.2, 0 m Na^+^, 140 mm K^+^, 143.5 mm Cl^−^; data of peak current recovery were fitted to a monoexponential function to obtain estimates of the recovery rate *k_r_. Inset*: The *k_r_* values obtained from the fits were plotted as function of voltage. *n* = 5–10. *C*, internal solution: pH 5.5, 0 m Na^+^, 0 m K^+^, 143.5 mm Cl^−^; the time course of peak current recovery was fitted to a monoexponential function to obtain estimates of the recovery rate *k_r_. Inset*: the *k_r_* values obtained from the fits were plotted as function of voltage. (*n* = 4–8). *D*, internal solution: pH 7.2, 0 m Na^+^, 0 m K^+^, 143.5 mm Cl^−^; data of peak-current recovery were fitted to a monoexponential function. *Inset*: *k_r_*-values obtained from the fits were plotted as function of voltage. (*n* = 4–12).

We repeated the experiments summarized in Fig. 6 in the presence of high [Cl^−^]*_i_* (143.5 mm) to differentiate between these two possibilities (*cf*. representative current traces in Fig. 8*A*). Regardless of the voltage, the recovery rate *k_r_* was not affected by high [Cl^−^]*_i_* in the presence of high [K^+^]*_i_* (Fig. 8*B*) or of high [H^+^]*_i_* (Fig. 8*C*). More importantly, increasing [Cl^−^]*_i_* in a K^+^-free solution at pH 7.2 enhanced the turnover rate of the transporter over the entire voltage range (Fig. 8*D*). These data rule out low affinity Cl_*i*_^−^ binding, but they are compatible with the hypothesis that Cl^−^ remains bound to SERT during the entire transport cycle.

## Discussion

Monoamine transporters can operate in both a forward transport and a substrate-exchange mode (3); in the substrate-exchange mode, the transporter returns from the inward-facing to the outward-facing conformation loaded with substrate and co-substrates. Thus, there ought to be a mechanism that safeguards against this futile cycling by promoting the forward-transport mode where the transporter returns to the outward-facing state without substrate. In addition, the transport reaction must be shielded from changes in membrane potential. Here, we show that these two requirements are met by the sequential release of Na^+^ from the inward-facing conformation and the binding of K^+^ (or H^+^). The pertinent key observations were: (*i*) Na^+^ dissociation from the inward-facing conformation of SERT was electrogenic, (*ii*) association of intracellular K^+^ or H^+^ was also electrogenic and blunted the voltage dependence of Na^+^ dissociation. and (*iii*) Cl^−^ did not affect the turnover rate of SERT, suggesting that it is bound to SERT during the entire catalytic cycle. Based on these observations, we propose that binding of K_*i*_^+^ (or of H_*i*_^+^) does not only increase the electrochemical driving force for uptake but also has a thus far unappreciated role under pre-steady-state conditions: the electrogenic association of K_*i*_^+^ (or of H_*i*_^+^) counteracts the electrogenicity of Na_*i*_^+^ release. This renders the progression through the transport-cycle voltage-independent.

### 

#### 

##### A Kinetic Model of SERT

**FIGURE 9.**
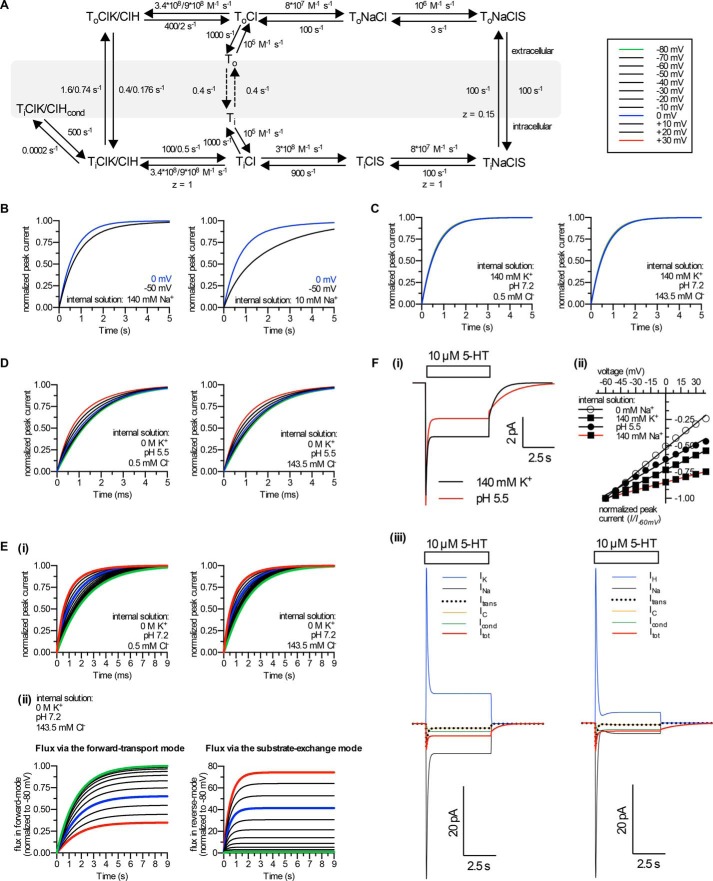
**A kinetic model of SERT.** *A*, scheme of the kinetic model. Intracellular cation binding carries one positive charge; *i.e.* Na_*i*_^+^ and K_*i*_^+^ or H_*i*_^+^ pass through the entire electric field while leaving/entering the intracellular binding site. The electrogenicity of Na_*i*_^+^-dissociation is instantaneously antagonized by association of K_*i*_^+^ or of H_*i*_^+^. SERT subsequently completes the transport cycle in a KCl- or HCl-bound state, which represents a voltage-independent rate-limiting step. Note that the return in a HCl-bound state is slower than the KCl-bound state. The conducting state is assumed to be in equilibrium with the KCl- or HCl-bound inward-facing conformation. *B*, induction of the substrate-exchange mode is voltage- and [Na^+^]*_i_*-dependent. Peak current recovery and its dependence on [Na^+^]*_i_* in K_*i*_^+^-free conditions at 0 mV and −50 mV was simulated. Reducing [Na^+^]*_i_* from 140 mm (*left panel*) to 10 mm (*right panel*) increases the voltage dependence of peak current recovery. This simulation reproduces data shown in Fig. 3, *C* and *D. C*, simulation of the turnover rate of SERT at a [K^+^]*_i_* of 140 mm at voltages from −80 to +30 mV. When a high [K^+^]*_i_* is used, the turnover-rate is voltage-independent. Increasing [Cl^−^]*_i_* from 500 μm (*left panel*) to 143.5 mm (*right panel*) does not affect the turnover rate. This simulation reproduces data shown in Figs. 6*C* and 8*B. D*, simulation of the turnover rate of SERT at a pH of 5.5 at 12 voltages (from −80 to +30 mV). The turnover rate of SERT is only slightly dependent on voltage at this [H^+^]*_i_*. Increasing [Cl^−^]*_i_* from 500 μm (*left panel*) to 143.5 mm (*right panel*) does not affect the turnover rate. This simulation reproduces data shown in Fig. 6*D* and 8*C. E*, *ii*, simulation of peak current recovery at a pH of 7.2 at 12 voltages (from −80 to +30 mV). Peak current recovery is voltage-dependent when a low [H^+^]*_i_* is used. Increasing [Cl^−^]*_i_* from 500 μm (*left panel*) to 143.5 mm (*right panel*) increases peak-current recovery. This simulation reproduces data shown in Fig. 6*B* and 8*D. ii*, the ratio of substrate-exchange to forward-transport mode increases with positive potentials because of higher relaxation via the substrate-exchange mode at positive potentials. *Flux* via the forward-transport mode was defined as ∫_0_^9^[ToCl]*dt*, because this state must be traversed in the forward-transport mode but is not met during the substrate-exchange mode. *Flux* via the substrate-exchange mode was defined as ∫_0_^9^[ToNaClS]/*dt*, because this state must be traversed in the substrate-exchange mode but is not met during the forward-transport mode. *F*, *i*, the model reproduces 5-HT-induced currents. Applying 10 μm 5-HT elicits an inwardly directed current composed of a peak and a steady-state current component. Increasing [H^+^]*_i_* in a K_*i*_^+^-free condition functionally replaces K_*i*_^+^, yet with a lower amplitude and slower relaxation kinetics. This can be accounted for by a slower transition rate from TiClH to ToClH than TiClK to ToClK (see *panel A*). This simulation reproduces data shown in Figs. 1*A*, 5B and 7A. *ii*, the peak current-voltage dependence is reduced to a similar extent as shown in Fig. 3*A* and 6*E* by increasing [Na^+^]*_i_*, [K^+^]*_i_*, or [H^+^]*_i_*. The data points were fitted by linear regressions. *iii*, dissection of the 5-HT-induced currents into the individual electrogenic partial reactions as function of time; the model shows that Na_*i*_^+^ dissociation induces a large inward current. This cannot be detected because a large outward current due to rapid K_*i*_^+^ binding eliminates it. Hence, the net current is substantially smaller than the current that is elicited by Na_*i*_^+^ dissociation. Note that the Na_*i*_^+^- and H_*i*_^+^-induced currents at pH 5.5 are smaller than the K_*i*_^+^- and Na_*i*_^+^-induced currents at pH 7.2/[K^+^]*_i_* of 140 mm; this is due to the higher turnover rate of the latter condition. *I_K_* and *I_H_*, capacitive current upon binding of K_*i*_^+^ and H_*i*_^+^, respectively; *I_Na_*, capacitive current upon dissociation of Na_*i*_^+^; *I*_trans_, capacitive current upon transition from outward- to inward-facing conformation; *I_c_*, sum of all capacitive currents; *I*_cond_, ionic current via the conducting state (TiClK/ClH_cond_, see *panel A*); *I*_tot_, resulting net current.

We tested the plausibility of these mechanistic interpretations by developing a kinetic model of SERT (Fig. 9*A*). This model reproduces our experimental data (Fig. 9, *B–F*). We stress that, for the sake of simplicity, the present model posits sequential binding of ions and substrate; it does not take into account the possibility of cooperative binding reactions because this was not tested in the present study.

Based on our results, we surmise that the full charge associated with the traversal of Na^+^ resides in the intracellular unbinding reaction. Our data suggest that this reaction dictates whether the transporter enters the forward-transport or substrate-exchange mode. We further tested this hypothesis by simulating the time- and voltage-dependent evolution of the different transporter modes (*i.e.* forward *versus* substrate exchange) during a peak current recovery experiment. Indeed, we observed that the transporter completed the catalytic cycle in the forward transport mode at negative membrane potentials but entered the substrate-exchange mode at positive potentials (Fig. 9*E*, *i* and *ii*). In addition, dissociation of intracellular Na^+^ after (simulated) 5-HT application evoked large inward currents (*cf. black traces* in Fig. 9*Fiii*). However, the ensuing (and rapid) association of K_*i*_^+^ or of H_*i*_^+^ is equally electrogenic and, due to the opposite signs, cancels out the electrogenicity of Na^+^ dissociation (*cf. blue traces* in Fig. 9*Fiii*). This renders transporter turnover voltage-independent (*cf*. Fig. 9, *C* and *D*), reduces the voltage dependence of the peak current (*cf*. Fig. 9*Fii*), and results in the small net current that is typically observed during a 5-HT challenge (*cf. red traces* in Fig. 9*Fiii*).

##### Conducting State of SERT

We incorporated an uncoupled Na^+^ conductance into the model. This conducting state has previously been shown to be in equilibrium with a K^+^-bound inward-facing conformation of SERT (13).

Our observation that intracellular H^+^ ions also support the steady-state current further corroborates the working model where a channel-like conducting state is occasionally formed during transition of the transporter from the inward-facing conformation to the outward-facing conformation (13, 33). Thus, our data are consistent with the assumption that the conducting state is in equilibrium with a KCl- or HCl-bound inward-facing conformation (*cf*. Fig. 9*A*).

##### Voltage-independent Rate-limiting Step

SERT is thought to display an electroneutral stoichiometry (11); our results are compatible with this conjecture. However, we emphasize that the stoichiometry of SERT remains to be elucidated, as we do not claim that we identified all electrogenic events during 5-HT uptake. For instance, we located the charge of one Na^+^ ion, but it is still not clear whether SERT transports one or two Na^+^ ions into the cell. It is, for instance, conceivable that both Na^+^ binding sites, which were visualized in the crystal structure of human SERT (34), are required for the transport of one single Na^+^ ion. In fact, a study with the Na_1_ site mutant SERT-N101A suggested that the Na_2_ site sufficed to drive substrate translocation provided the Na_1_ site was occupied by non-permeant Ca^2+^ (35).

Regardless of whether SERT exhibits an electrogenic or electroneutral stoichiometry, our data show that in pre-steady-state conditions SERT is tuned to operate in a voltage-independent fashion. Given the firing pattern of serotonergic neurons (*e.g.* approximately 2–15 action potentials s^−1^ in macaques; Ref. 36), its relatively low turnover rate (approximately 1.6 s^−1^ at room temperature), and because it acts in a relay with the vesicular monoamine transporter (6), it is unlikely that SERT ever reaches steady-state *in vivo*. It may thus be more important for 5-HT uptake to assure progression to a voltage-independent rate-limiting step than displaying an electroneutral stoichiometry. In fact, when employing the same experimental conditions that supported the steady-state current, Mager *et al.* (10) observed voltage-independent substrate uptake. Here, we show that this is achieved by (*i*) electrogenic binding of intracellular K^+^ or H^+^, which cancels out the electrogenic nature of intracellular Na^+^ release, and (*ii*) by the negative charge of a Cl^−^ ion. To account for these features in the model, Na^+^, K^+^, and H^+^ must carry the same charge (*cf*. Fig. 9, *A* and *Fiii*). This indicates that these ions have highly overlapping, if not the same, binding sites. A recent study (37) suggests that K^+^ binds to the Na_2_ site of LeuT, the site that is thought to gate intracellular substrate release in the LeuT superfamily (17–24). Interestingly, this requires a negatively charged Glu-290, the carboxylate of which corresponds to bound Cl^−^ in the eukaryotic transporters (37). A recent study indicates that intracellular K^+^ precludes Na^+^ rebinding to the inward facing conformation of LeuT (38). Our data are consistent with this sequence of events and together with other studies (3, 4, 39) contribute to the emerging concept that antiport of K^+^ or of H^+^ is a key feature in the transport cycle of the LeuT superfamily. This model also posits that the negative charge provided either by Cl^−^ or by a glutamate residue remains available during the entire catalytic cycle. It has been a matter of debate, whether the electrochemical potential of Cl^−^ serves as an energy source to drive transport by members of the LeuT superfamily. The electrochemical driving force for substrate uptake is the sum of the electrochemical potentials of all (co-)substrates involved in the transport stoichiometry (40). The reversal potential of Cl^−^ is approximately −70 mV, which is close to the typical resting membrane potential of neurons. It is thus questionable whether the Cl^−^ gradient contributes any energy to substrate uptake. It is nevertheless clear that Cl^−^ is required to establish an interaction network, which allows for substrate binding and translocation (41).

##### Amphetamines and the Substrate-exchange Mode

Finally, the present findings are relevant to understand amphetamine-induced monoamine release (6). Amphetamines are exogenous substrates of the monoamine transporters. Hence, upon binding to the outward-facing conformation, they are translocated into the cell and subsequently dissociate from the inward-facing conformation. This presents a binding site to intracellular substrate (*e.g.* 5-HT) that can then be translocated out of the cell. Hence, amphetamines switch monoamine transporters into a substrate-exchange mode (6). Amphetamine-induced monoamine release is enhanced by increasing intracellular [Na^+^] (6, 42). Moreover, amphetamine-induced dopamine release increases with positive membrane potentials (42). The present data suggest that this voltage dependence is (at least in part) attributable to voltage-dependent binding of intracellular Na^+^ to the inward-facing conformation of the cognate monoamine transporter.

## Experimental Procedures

### 

#### 

##### Whole-cell Patch Clamp

Patch clamp recordings were performed with HEK-293 cells stably expressing human SERT. In all instances, the cells were seeded at low density 24 h before measuring currents. Substrate-induced human SERT currents were recorded under voltage clamp using the whole-cell patch clamp technique. Internal solutions were as follows. The Na^+^- and K^+^-free internal solution comprising 143.5 mm Cl^−^ contained 10 mm HEPES, 1 mm CaCl_2_, 0.7 mm MgCl_2_, 10 mm EGTA, and 140 mm NMDGCl and was titrated to a pH of 7.2 using NMDG. For solutions used in the experiments shown in Figs. 2 and 3, the value of the titrated Na^+^ concentration was subtracted from the NMDGCl fraction, thereby maintaining constant osmolality: *e.g*. 10 mm NaCl plus 130 mm NMDGCl. For experiments using a high K^+^ concentration, 140 mm KCl was used instead of NMDGCl or NaCl.

1 ml of the Na^+^- and K^+^-free internal solution comprising 500 μm Cl^−^ contained 3.5 μl of the solution comprising 143.5 mm Cl^−^ (described above) and 996.5 μl of a Cl^−^-free solution. The Cl^−^-free solution contained 10 mm HEPES, 1 mm calcium methanesulfonate), 0.7 mm magnesium acetate, 10 mm EGTA, and 140 mm NMDG methanesulfonate and was titrated to pH 7.2 with NMDG. For experiments using a high K^+^ concentration, 140 mm potassium methanesulfonate was used instead.

Internal solutions with a pH of 5.5 contained 10 mm MES buffer, 1 mm CaCl_2_, 0.7 mm MgCl_2_, 10 mm EGTA, and 140 mm NMDGCl and were titrated to pH 5.5 with NMDG. Adjustments of the Cl^−^ concentration were made in a manner similar to that described for the solutions at pH 7.2 (see above). The liquid junction potential was below 1 mV for all conditions used in this study. Hence, it was not necessary to correct for liquid junction potentials.

The cells were continuously superfused with external solution (143 mm NaCl, 2.5 mm CaCl_2_, 2 mm MgCl_2,_ 20 mm glucose, and 10 mm HEPES adjusted to pH 7.4 with NaOH). Currents were recorded at room temperature (20–24 °C) using an Axopatch 200B amplifier and pClamp 10.2 software (MDS Analytical Technologies). Unless otherwise indicated, the washout period (between sweeps) after 5-HT application was 30 s. Current traces were filtered at 1 kHz and digitized at 2 kHz using a Digidata 1320A (MDS Analytical Technologies). Drugs were applied using a DAD-12 (Adams & List, Westbury, NY), which allows for complete solution exchange around the cells within 100 ms. Current amplitudes in response to 5-HT application were quantified using Clampfit 10.2 software. Passive holding currents were subtracted, and the traces were filtered using a 100-Hz digital Gaussian low-pass filter.

##### Statistics

Uncertainties are shown as 95% confidence intervals (*square brackets* in the legends). Non-overlapping 95% confidence intervals (in figure legends) were considered as significant differences.

##### Modeling

We developed a kinetic model of SERT based on a published model (13) that was modified to account for our data (*cf*. Fig. 9*A*). The time-dependent changes in state occupancies were evaluated by numerical integration of the resulting system of differential equations using Systems Biology Toolbox and Matlab 2015a (Mathworks). The voltage dependence of individual rates was modeled according to Laüger (43) assuming a symmetric barrier as *k_ij_* = *k*^0^*_ij_*exp(−*z_Qi,j_FV*/2*RT*), with *F* = 96,485 coulombs·mol^−1^, *R* = 8.314 JK^−1^mol^−1^, *V* is the membrane voltage in volts, and *T* = 293 K. Coupled membrane currents in response to substrate application were calculated as *I* = (−*F*Σ*z_Q,ij_*(*p_i_k_ij_* − *p_j_k_ji_*))*NC*/*N*_A_, where *z_Q,ij_* is the net charge transferred during the transition, *NC* is the number of transporters set to 4 × 10^6^, and *N*_A_ if 6.022 × 10^23^. The uncoupled current was modeled as a current through a Na^+^-permeable channel with *I* = *P*_c_γ*NC*(*V* − *V*_rev_), where *P*_c_ is the occupancy of the channel state, γ is the single-channel conductance of 2.4 picosiemens, *NC* is the number of channels (4 × 10^6^), *V* is the membrane voltage, and *V*_rev_ is the reversal potential of Na^+^ at 80 mV. The extra- and intracellular ion concentrations were set to the values used in patch clamp experiments. To account for the non-instantaneous onset of substrate in the patch clamp experiments, substrate application was modeled as exponential rise with a time constant of 10 ms. The time course of recovery after substrate application was modeled as the time course of return to ToClNa (*cf*. Fig. 9*A*).

## Author Contributions

P. S. H., M. F., and W. S. conceptualized the study and designed the experiments. P. S. H. and W. S. designed the model. P. S. H. performed all experiments and analyzed the data. P. S. H., M. F., and W. S. wrote the manuscript.

## Footnotes

2 The abbreviations used are:

SLC: solute carrier
5-HT: 5-hydroxytryptamine
DAT: dopamine transporter
GAT1: GABA transporter 1
LeuT: leucine transporter
MES: methanesulfonate
NMDG: *N*-methyl-d-glucamine
NMDGCl: NMDG chloride
SERT: serotonin transporter.
